# Impact of easing COVID-19 lockdown restrictions on traumatic injuries in Riyadh, Saudi Arabia: one-year experience at a major trauma centre

**DOI:** 10.1186/s12889-023-14981-9

**Published:** 2023-01-04

**Authors:** Rayan Jafnan Alharbi, Rami Al-Jafar, Sharfuddin Chowdhury, Muhammad Aziz Rahman, Ateeq Almuwallad, Abdullah Alshibani, Virginia Lewis

**Affiliations:** 1 Department of Emergency Medical Service, College of Applied Medical Sciences, Jazan University, 45142, Al Maarefah Rd, Jazan, Saudi Arabia; 2 Department of Epidemiology and Biostatistics, School of Public Health, Imperial College London, London, UK; 3 Department of Data Services, Lean Business Services, Riyadh, Saudi Arabia; 4 Trauma Center, King Saud Medical City, Riyadh, Saudi Arabia; 5 School of Health, Federation University Australia, Berwick, VIC Australia; 6 Centre for Trauma Science, Blizard Institute Queen Mary University, London, UK; 7 Department of Emergency Medical Services, College of Applied Medical Sciences, King Saud bin, Abdulaziz University for Health Sciences, Riyadh, Saudi Arabia; 8 King Abdullah International Medical Research Center, Riyadh, Saudi Arabia; 9 Australia Institute for Primary Care and Ageing (AIPCA), La Trobe University, Melbourne, VIC Australia

**Keywords:** Covid-19, Pandemic, Easing restrictions, Injury, Major trauma, Motor Vehicle Crashes

## Abstract

**Introduction:**

Lockdown restrictions due to the COVID-19 pandemic have reduced the number of injuries recorded. However, little is known about the impact of easing COVID-19 lockdown restrictions on the nature and outcome of injuries. This study aims to compare injury patterns prior to and after the easing of COVID-19 lockdown restrictions in Saudi Arabia.

**Method:**

Data were collected retrospectively from the Saudi TraumA Registry for the period between March 25, 2019, and June 21, 2021. These data corresponded to three periods: March 2019–February 2020 (pre-restrictions, period 1), March 2020–June 2020 (lockdown, period 2), and July 2020–June 2021 (post easing of restrictions, period 3). Data related to patients’ demographics, mechanism and severity of injury, and in-hospital mortality were collected and analysed.

**Results:**

A total of 5,147 traumatic injury patients were included in the analysis (pre-restrictions *n* = 2593; lockdown *n* = 218; post easing of lockdown restrictions *n* = 2336). An increase in trauma cases (by 7.6%) was seen in the 30–44 age group after easing restrictions (*n* = 648 vs. 762, *p* < 0.01). Motor vehicle crashes (MVC) were the leading cause of injury, followed by falls in all the three periods. MVC-related injuries decreased by 3.1% (*n* = 1068 vs. 890, *p* = 0.03) and pedestrian-related injuries decreased by 2.7% (*n* = 227 vs. 143, *p* < 0.01); however, burn injuries increased by 2.2% (*n* = 134 vs. 174, *p* < 0.01) and violence-related injuries increased by 0.9% (*n* = 45 vs. 60, *p* = 0.05) post easing of lockdown restrictions. We observed an increase in in-hospital mortality during the period of 12 months after easing of lockdown restrictions—4.9% (114/2336) compared to 12 months of pre-lockdown period—4.3% (113/2593).

**Conclusion:**

This is one of the first studies to document trauma trends over a one-year period after easing lockdown restrictions. MVC continues to be the leading cause of injuries despite a slight decrease; overall injury cases rebounded towards pre-lockdown levels in Saudi Arabia. Injury prevention needs robust legislation with respect to road safety measures and law enforcement that can decrease the burden of traumatic injuries.

**Supplementary Information:**

The online version contains supplementary material available at 10.1186/s12889-023-14981-9.

## Introduction

Coronavirus disease 2019 (COVID-19) was discovered in December 2019 in Wuhan, China [1]. Since then, the virus has spread rapidly worldwide. On January 30, 2020, the World Health Organization (WHO) announced the outbreak as a public health emergency of international concern and declared it a global pandemic on March 11, 2020 [2]. This outbreak of COVID-19 has forced many countries worldwide to enforce societal lockdowns and policies to contain the rapid infection spread. In Saudi Arabia, the first case of COVID-19 was reported on March 2, 2020, and cases soon increased exponentially [3]. The government of Saudi Arabia initiated restrictions from early March 2020; such restrictions included suspension of schools, universities, social events and international flights. Furthermore, on April 6, 2020, a stay at home order was introduced in the capital city of Saudi Arabia, Riyadh [3]. On June 21, 2020, the lockdown restrictions were lifted from all regions across the country and life returned to near-normal, with adherence to health instructions and maintenance of social distancing [3].

Traumatic injury is a leading cause of death and disability worldwide [4, 5]. The COVID-19 pandemic and the government’s restriction policies designed to fight this pandemic have undoubtedly affected the incidence of injury. For example, recent evidence showed that mandated lockdown restrictions resulted in significant reduction in injury-related road trauma by 42.6%, but increased household and assault injuries by 9.9% and 7%, respectively [6–8]. In Saudi Arabia, traumatic injuries, particularly injuries arising from motor vehicle crashes (MVC), are a major public health problem [9–12]. A recently published study reported a significant reduction in injuries by 26.8% during the country-wide lockdown [13]. A reduction in injury incidence was also seen in other countries such as Australia [14], Ireland [15], New Zealand [16] and South Africa [17].

There are limited studies describing the impact of lifting COVID-19 lockdown restrictions on traumatic injuries. It is anticipated that injury cases would rebound towards pre-lockdown levels because people will travel more and practice sports after lockdowns end. The patterns and outcome of injuries after easing lockdown restrictions in the Middle East, and specifically in Saudi Arabia, are unknown. This study aims to assess the impact of easing COVID-19 lockdown restrictions on traumatic injuries in Saudi Arabia.

## Materials and methods

Data were extracted retrospectively from King Saud Medical City (KSMC) trauma registry known as the Saudi TraumA Registry (STAR). KSMC is located in Riyadh and is one of the few major trauma centres in Saudi Arabia with a bed capacity of 1,400. Approximately 350 visits are recorded per day to the emergency department (ED) and more than 300 trauma cases are seen per month [18]. The registry collects data of injury patients meeting any of the following criteria:

- 1. Principal diagnosis of injury, and
- 2. Death in the ED after injury, or
- 3. Inpatient admission ≥ 3 calendar days, or
- 4. Inpatient death following injury, or
- 5. Admission to the Intensive Care Unit (ICU)

This study included all traumatic injury patients who met the KSMC registry criteria and presented to the ED or admitted to the hospital between March 25, 2019, and June 21, 2021. These data correspond to three periods: March 25, 2019–March 24, 2020 (pre-restrictions, period 1, 12 months), March 25, 2020–June 21, 2020 (lockdown, period 2, 3 months) and June 22, 2020–June 21, 2021 (post easing of restrictions, period 3, 12 months). Data regarding injury events and patients’ characteristics such as demographics, time and place of injury, mechanism and severity of injury, definitive care mode of arrival were extracted from the STAR. Other variables included prehospital and in-hospital vital signs such as systolic blood pressure (SBP), heart rate (HR) and respiratory rate (RR). Furthermore, ED and in-hospital outcomes were included in the data analyses.

Although the focus of this study is comparing one year of injury data before and after easing of the lockdown restrictions, our data was extracted continuously from March 2019 to June 2021, including a three-month lockdown period. Therefore, injury data during the lockdown phase were included in this study as phase 2 (March 25, 2020–June 21, 2020).

### Statistical analyses

Statistical analyses were performed using the R program for statistical computing, version R-4.1.1. Frequencies and percentages in each time period and for the total study population are reported for categorical variables such as age group, mechanism of injury, place of injury, type of injury and mode of arrival. Continuous variables such as prehospital and in-hospital vital signs are reported with mean and standard deviation (SD) or with median and interquartile range where appropriate. ICU admissions and in-hospital mortality are presented as frequencies and percentages. The significance of changes in variable distributions and/or means between periods 1 (pre restrictions) and 3 (post easing of restrictions), Chi-square test was used and the Student's t-test for independent samples or Mann–Whitney U test for continuous variables as appropriate. The three-month lockdown data are only presented as descriptive data; the comparison of injury characteristics, pattern, volume and outcome focused more on pre and post easing of COVID-19 restrictions. We did not focus on the lockdown phase because this period was previously studied in Riyadh, Saudi Arabia [13].

In this study, univariate analyses were performed to identity predictors of in-hospital mortality for time periods 1 and 3. Factors associated significantly with the dependent variable were entered into a multivariable logistic regression model. Furthermore, multivariable logistic regression modelling was also used to predict ICU admissions (dependent variable (DV)) by the mechanism of injury (including motor vehicle, motorcycle/pedal cyclist, pedestrian, fall, assault, burn and penetrating—independent variables (IV)) for periods 1 (pre restrictions) and 3 (post easing of restrictions). The two regression models are presented individually for the periods of pre and post easing of restrictions and adjusted for covariates including age and gender. The adjusted odds ratios (AOR) and 95% confidence intervals (CIs) were calculated with respect to the likelihood of difference between periods 1 and 3. The level for statistical significance was a *p* value of < 0.05 in all analyses. This study was approved by the Institutional Review Board Committee at King Saud Medical City (H1RI-20–June21-01).

## Results

### Demographic and clinical characteristics

A total of 5,147 traumatic injury patients were admitted over the study period: March 25, 2019–June 21, 2021. During the periods that were investigated, of the 5,147 patients; 2,593 (50.3%) patients were in the pre-restrictions group (period 1), 218 (4.3%) patients were in the lockdown group (period 2), and 2,336 (45.4%) patients were in post easing of lockdown restrictions group (period 3). Table 1 shows the characteristics of the study population in each of the three time periods and overall. Tests of significance of change were only undertaken for comparisons of periods 1 and 3.

**Table 1:** Demographic and presentation characteristics of traumatic injury patients of pre-restrictions, restrictions/lockdown, and after easing the restrictions

Variables	Total	Pre-restrictions	Lockdown^$^	After easing restrictions	% changes	*P* value
		Period 1 12 months	Period 2 3 months	Period 3 12 months	**P1 to P3**	
	*N* = 5147	*N* = 2593	*N* = 218	*N* = 2336	-5.2	
Mean age in years (SD)	33 (17.5)	32.6 (17.6)	32 (16.2)	34 (17.5)		
Age group, *n* (%)	**5147**	**2593**	**218**	**2336**		
0–14	462 (9)	252 (9.7)	22 (10.1)	188 (8)	-1.7	0.05*
15–29	2097 (40.7)	1160 (44.7)	77 (35.3)	860 (36.8)	-7.9	< 0.01*
30–44	1488 (28.9)	648 (25)	78 (35.7)	762 (32.6)	+ 7.6	< 0.01*
45–59	631 (12.3)	302 (11.6)	24 (11)	305 (13.1)	+ 1.5	0.14
≥ 60	469 (9.1)	231 (8.9)	17 (7.8)	221 (9.5)	+ 0.6	0.53
Gender *n* (%)	**5147**	**2593**	**218**	**2336**		
Male	4337 (84.3)	2170 (83.7)	190 (87.1)	1977 (84.6)	+ 0.9	0.38
Female	810 (15.7)	423 (16.3)	28 (12.8)	359 (15.4)	-0.9	
Injury time, *n* (%)	**2540**	**1335**	**99**	**1106**		
AM	1138 (44.8)	588 (44)	43 (43.4)	507 (45.8)	+ 1.8	0.39
PM	1402 (55.2)	747 (56)	56 (56.6)	599 (54.2)	-1.8	
Mechanism of injury, *n* (%)	**5147**	**2593**	**218**	**2336**		
Motor Vehicle	2043 (39.7)	1068 (41.2)	85 (38.9)	890 (38.1)	-3.1	0.03*
Motorcycle	182 (3.5)	91 (3.5)	1 (0.5)	90 (3.9)	+ 0.4	0.57
Pedal cyclist	20 (0.4)	7 (0.3)	3 (1.4)	10 (0.4)	+ 0.1	0.48
Pedestrian	381 (7.4)	227 (8.8)	11 (5)	143 (6.1)	-2.7	< 0.01*
Fall > 1 m	827 (16.1)	384 (14.8)	40 (18.3)	403 (17.3)	+ 2.5	0.02*
Fall ≤ 1 m	832 (16.2)	435 (16.8)	30 (13.8)	367 (15.7)	-1.1	0.33
Assault/violence	107 (2.1)	45 (1.7)	2 (0.9)	60 (2.6)	+ 0.9	0.05*
Burn	333 (6.5)	134 (5.2)	25 (11.5)	174 (7.4)	+ 2.2	< 0.01*
Accidental	260 (5.1)	121 (4.7)	12 (5.5)	127 (5.4)	+ 0.7	0.24
Penetrating	154 (2.9)	77 (2.9)	9 (4.1)	68 (2.9)	0	0.97
Other	8 (0.2)	4 (0.2)	0 (0)	4 (0.2)	0	
Injury place n (%)	**3367**	**1742**	**124**	**1501**		
Farm	25 (0.7)	19 (1.1)	0 (0)	6 (0.4)	-0.7	0.03*
Home	583 (17.3)	252 (14.5)	22 (17.7)	309 (20.6)	+ 6.1	< 0.01*
Industrial	111 (3.3)	64 (3.7)	2 (1.6)	45 (3)	-0.7	0.23
School	9 (0.3)	7 (0.4)	0 (0)	2 (0.1)	-0.3	0.23
Road	2606 (77.4)	1379 (79.1)	100 (80.6)	1127 (75.1)	-4	< 0.01*
Sports	33 (1)	21 (1.2)	0 (0)	12 (0.8)	-0.4	0.27
Injury type, *n* (%)	**9582**	**4891**	**430**	**4261**		
Head	1350 (14.1)	707 (14.5)	66 (15.3)	577 (13.5)	-1	0.04*
Face	939 (9.8)	492 (10.1)	46 (10.7)	401 (9.4)	-0.7	0.11
Neck	33 (0.3)	19 (0.4)	1 (0.2)	13 (0.3)	-0.1	0.55
Thorax	1235 (12.9)	631 (12.9)	63 (14.6)	541 (12.7)	-0.2	0.35
Abdomen & pelvic	461 (4.8)	243 (4.9)	17 (4)	201 (4.7)	-0.2	0.37
Spine	1597 (16.7)	773 (15.8)	76 (17.7)	748 (17.6)	+ 1.8	0.09
Upper extremities	1306 (13.6)	694 (14.2)	50 (11.6)	562 (13.2)	-1	0.03*
Lower extremities	2246 (23.4)	1135 (23.2)	86 (20)	1025 (24.1)	-0.9	0.96
Other trauma	415 (4.3)	197 (4)	25 (5.8)	193 (4.5)	+ 0.5	0.42
Prehospital physiological assessment, mean (SD)						
First systolic BP	124.8 (21.6)	125.2 (21.8)	122.3 (22.9)	124.5 (21.3)		0.59
First heart rate	93.1 (18.3)	94.7 (19.2)	93.6 (18.2)	91 (1)		< 0.01*
First RR	17.8 (3.7)	18.1 (4)	18.2 (3.2)	17.3 (3.4)		< 0.01*
Definitive Care Mode of Arrival, n (%)	**4456**	**2268**	**183**	**2005**		
Red Crescent ambulance	1272 (28.5)	632 (27.9)	46 (25.1)	594 (29.6)	+ 1.7	0.41
Private ambulance	87 (1.9)	58 (2.5)	4 (2.2)	25 (1.2)	-1.3	< 0.01*
Government ambulance	2032 (45.6)	963 (42.5)	105 (57.4)	964 (48.1)	+ 5.6	< 0.01*
Helicopter	52 (1.2)	35 (1.5)	0 (0)	17 (0.8)	-0.7	0.05*
Private/police vehicle	1013 (22.7)	580 (25.6)	28 (15.3)	405 (20.2)	-5.4	
Trauma Team Activation,* n* (%)	**5126**	**2583**	**217**	**2326**		
Yes	358 (7)	221 (8.6)	26 (12)	111 (4.8)	-3.8	< 0.01*
No	4768 (93)	2362 (91.4)	191 (88)	2215 (95.2)	+ 3.8	< 0.01*
Blood transfusion in ED, *n* (%)	**5139**	**2591**	**217**	**2331**		
Yes	192 (3.7)	112 (4.3)	11 (5.1)	69 (2.9)	-1.4	0.02*
No	4947 (96.3)	2479 (95.7)	206 (94.9)	2262 (97.1)	-1.4	
On arrival at the ED, mean (SD)						
First systolic BP	126.4 (21.8)	126.8 (22.7)	126.5 (22.3)	126 (20.8)	-1.9	0.23
First heart rate	94 (19.2)	94.3 (19.8)	94.7 (20.7)	93.7 (18.4)	-1.4	0.22
First RR	19.9 (3)	20 (3)	19.9 (2.8)	19.8 (2.9)	-0.1	0.20
First O_2_ saturation	98 (2) ^Μ^	98 (3) ^Μ^	98 (2) ^Μ^	98 (2) ^Μ^		
Respiratory assistance, *n* (%)	**5052**	**2533**	**215**	**2304**		
Assisted respiration	768 (15.2)	445 (17.6)	33 (15.3)	290 (12.6)	-5	< 0.01*
Unassisted respiration	4284 (84.8)	2088 (82.4)	182 (85.1)	2014 (87.4)	+ 5	< 0.01*

Period 1 = March 25, 2019 – March 24, 2020; Period 2 = March 25 – June 21, 2020; Period 3 = June 22, 2020 – June 21, 2021

*IQR* Interquartile Range, *BP* Blood Pressure, *RR* Respiratory Rate

% changes = change in value After easing restrictions compared to Pre-restrictions

*P*-value: Represent a calculation between period 1 and 3 groups

^*^Significant *P*-value

^Μ^Median

^$^The lockdown column is just there for completeness

Comparing period 1 (pre restrictions, 12 months) and period 3 (post easing of restrictions, 12 months), there was a reduction in the total number of trauma cases by 5.2% post easing of restrictions. There were also changes in the distribution of cases by age group; we observed a reduction of 1.7% with respect to the proportion of trauma cases in the age group of < 14 years (*n* = 252 (9.7%) vs 188 (8%), *p* = 0.05), and of 7.9% with respect to the age group of 15–29 years (*n* = 1160 (44.7%) vs 860 (36.8%), *p* < 0.01); however, a significant increase of 7.6% was seen in those aged 30–44 years, post easing of restrictions (*n* = 648 (25%) vs 762 (32.6%), *p* < 0.01). The post easing of restrictions group saw a reduction in trauma team activation by 3.8% (*n* = 221 (8.6%) vs 111 (4.8%), *p* < 0.01). There was no significant difference between the numbers of prehospital and in-hospital physiological assessments conducted in the ED or hospital admissions between the two periods; however, the proportion of patients requiring respiratory assistance decreased by 5% post lockdown (*n* = 445 (17.6%) vs 290 (12.6%), *p* < 0.01). Incidences of private/police vehicles as modes of arrival decreased by 5.4% (*n* = 580 (25.6%) vs 405 (20.2%), *p* < 0.01) post lockdown. We observed a change in the type of injury, including a reduction in head injury by 1% (*n* = 707 (14.5%) vs 577 (13.5%), *p* = 0.04) and injury to upper extremities by 1% (*n* = 694 (14.2%) vs 562 (13.2%), *p* = 0.03).

### Mechanism of injury

**Fig. 1:**
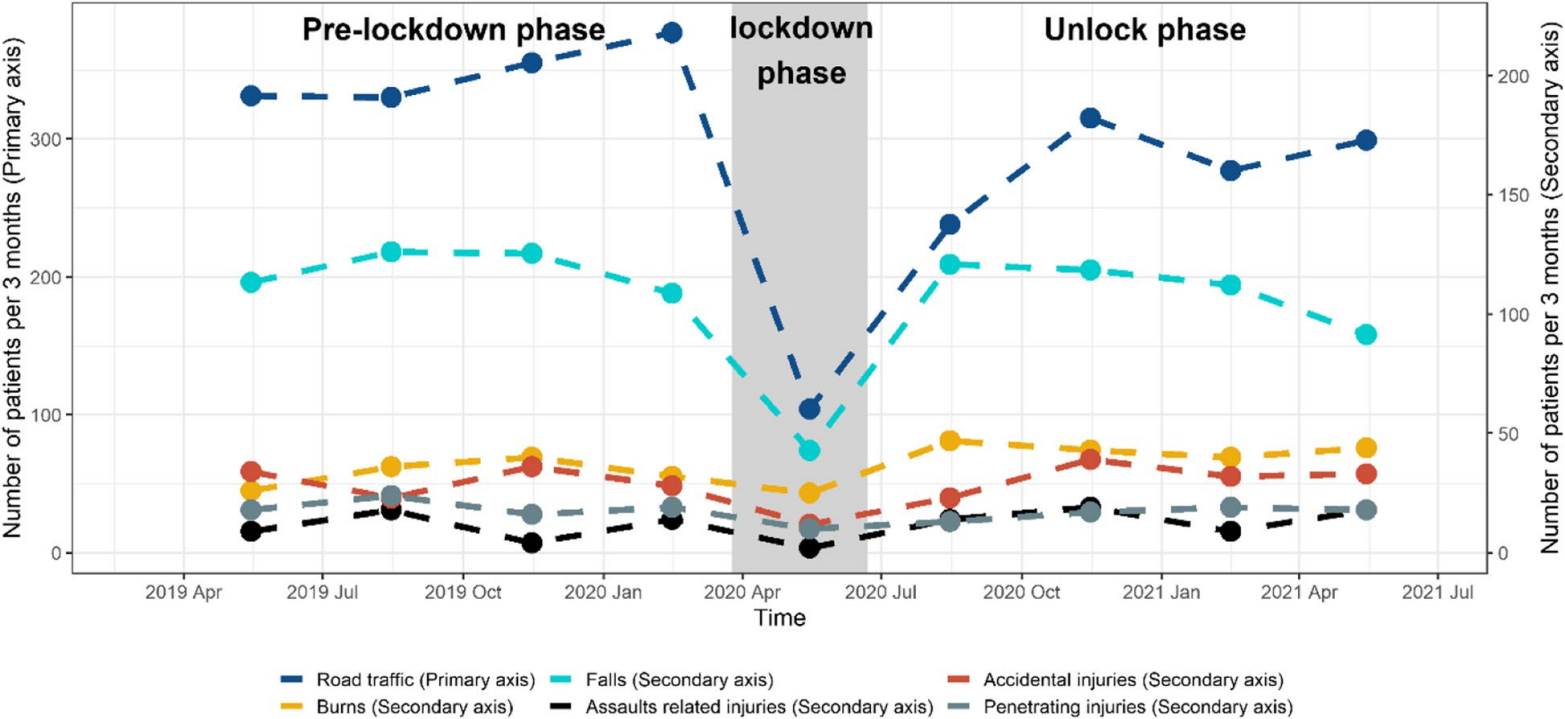
The mechanism of injury across the three periods

**Fig. 2:**
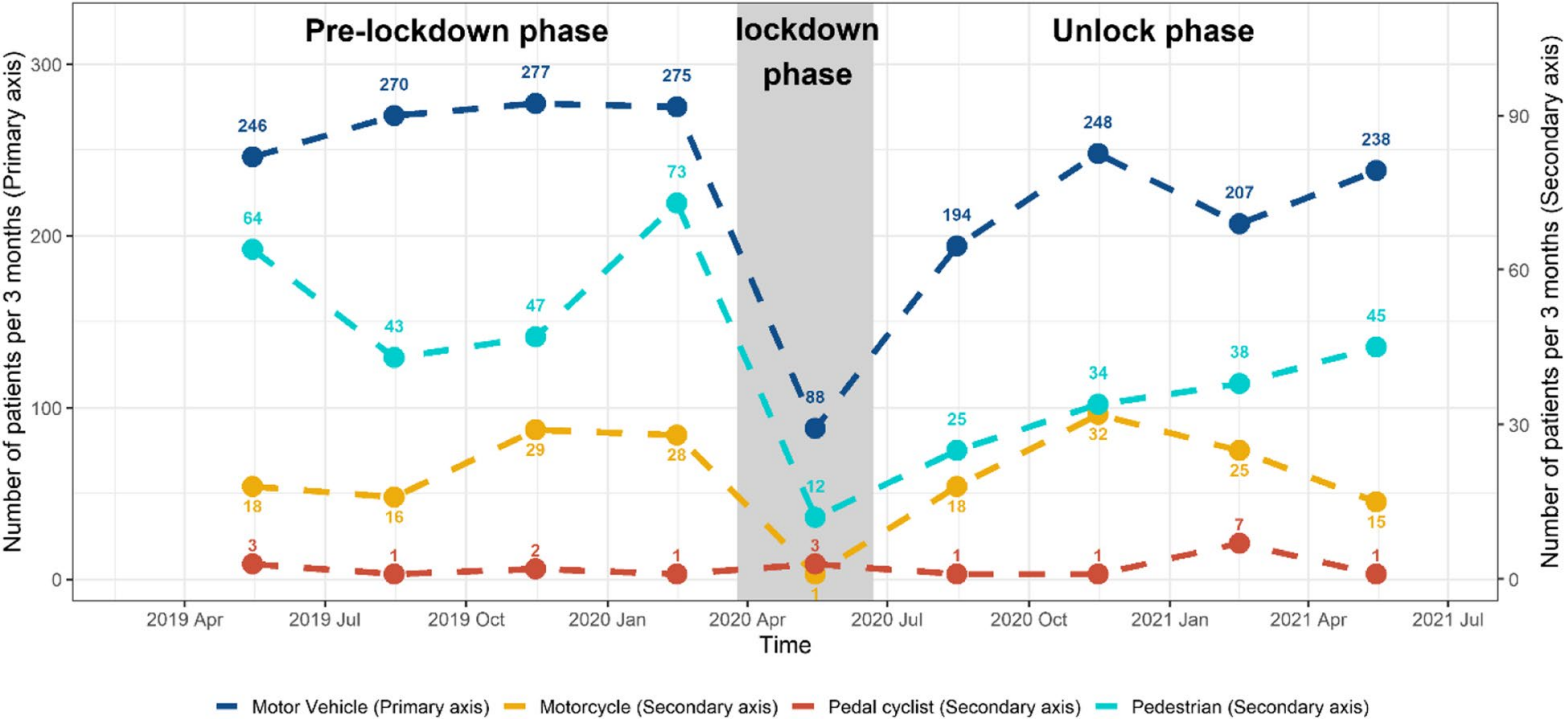
The mechanism of injury by road trauma across the three periods

Overall, 1,402 (55.2%) injury events occurred in PM time, while 1,138 (44.8%) injury cases occurred in AM time. The definition of PM and AM is referring to clock times between noon and midnight and midnight to noon, respectively. The main mechanism of injury over the whole study period was MVC (*n* = 2043, 39.7%), followed by falls (*n* = 1659, 32.3%). Comparing injury mechanisms between pre and post lockdown periods, incidences of MVC-related injury reduced by 3.1% (*n* = 1068 (41.2%) vs 890 (38.1%), *p* = 0.03) and pedestrian injury decreased by 2.7% (*n* = 227 (8.8%) vs 143 (6.1%), *p* < 0.01). However, burn and home-related injuries increased by 2.2% (*n* = 134 (5.2%) vs 174 (7.4%), *p* < 0.01) and 6.1% (*n* = 252 (14.5%) vs 309 (20.6%), *p* < 0.01), respectively, after easing of lockdown restrictions. There was an increase of 2.5% in fall injuries > 1 m (*n* = 384 (14.8%) vs 403 (17.3%), *p* = 0.02) and a rise of 0.9% in assault/violence-related injuries (*n* = 45 (1.7%) vs 60 (2.6%), *p* = 0.05) post easing of lockdown restrictions (Figs. 1 and 2).

### Patient outcomes

**Fig. 3:**
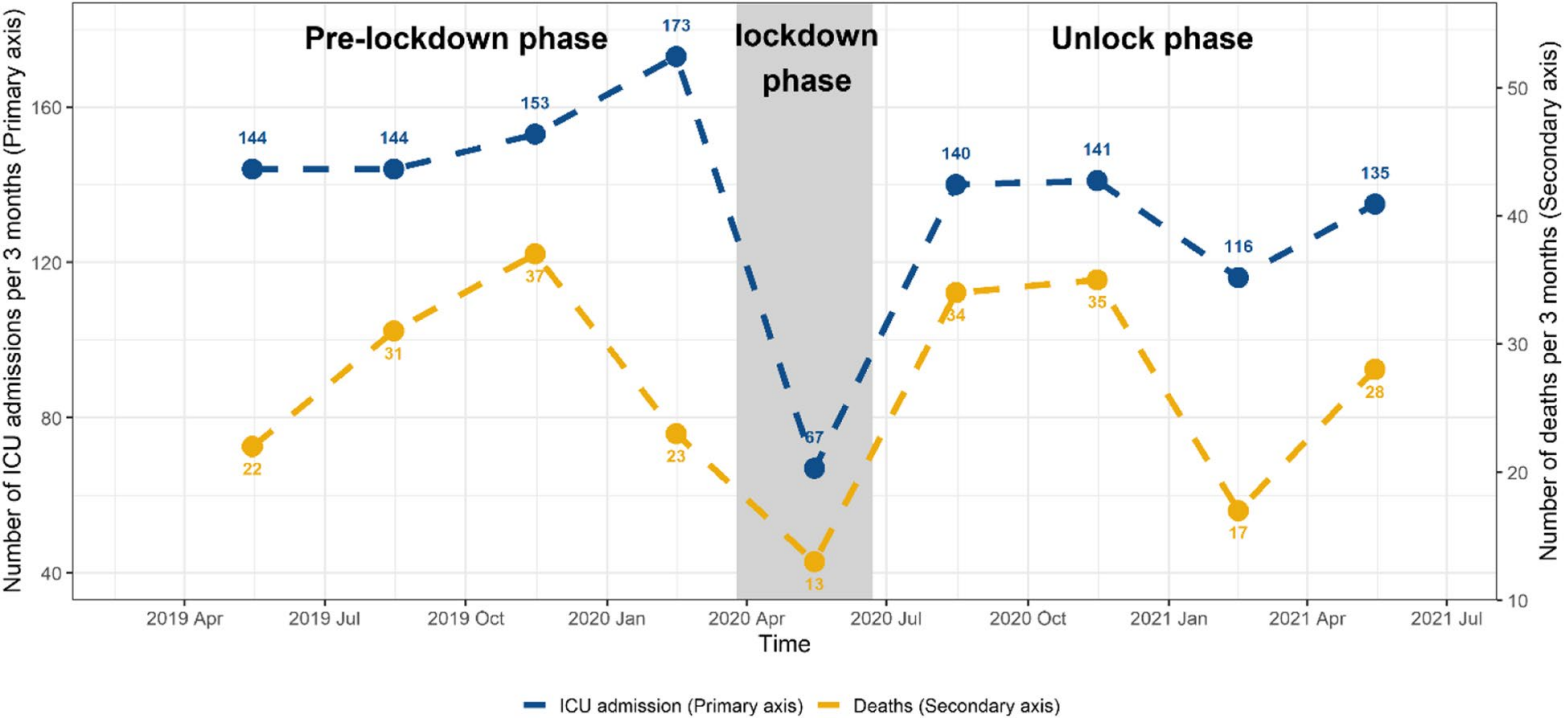
The number of ICU admissions and deaths across the three periods

The median injury severity score (ISS) was nine, and ICU admission was seen in 1,213 cases (23.6% of the study population) (Fig. 3). There was a reduction in cases of low level of consciousness, indicated by a Glasgow Coma Scale (GCS) score of 3 to 8 by 3.7% (*n* = 245 (13.1%) vs 174 (9.4%), *p* < 0.01) and a reduction in the severity of injury (ISS > 40) by 0.7% (*n* = 42 (1.6%) vs 21 (0.9%), *p* = 0.03) after lifting of the lockdown restrictions. Transfer of patients from the ED to the operating theatre reduced by 2.2% (*n *= 137 (5.3%) vs 72 (3.1%), *p* < 0.01) after easing of the lockdown restrictions. There was no change in the length of ICU stay between the two periods (median was nine days); however, the length of hospital stay (LOHS) increased by a day compared with pre restrictions (median was eight days compared to nine days for the post lifting of restrictions group). The overall in-hospital mortality was 4.7% (240/5147). We observed an increase in in-hospital mortality in the 12 months after easing of lockdown restrictions compared to the 12 months before the lockdown, i.e., 4.9% (114/2336) and 4.3% (113/2593) *p* = 0.42, respectively. Table 2 shows patient outcomes following injury events.

**Table 2:** Level of consciousness, injury severity, and patient outcome following injury events

Variables and outcomes	Total	Pre-restrictions	Lockdown^$^	After easing restrictions	% changes P1 to P3	*P* value
		Period 1 12 months	Period 2 3 months	Period 3 12 months		
	*N *= 5147	*N* = 2593	*N* = 218	*N* = 2336	-5.2	
GCS score, *n* (%)	**3869**	**1866**	**142**	**1861**		
13 − 15	3325 (85.9)	1553 (83.2)	117 (82.4)	1655 (88.9)	+ 5.7	< 0.01*
9 − 12	106 (2.7)	68 (3.6)	6 (4.2)	32 (1.7)	-1.9	< 0.01*
3 − 8	438 (11.3)	245 (13.1)	19 (13.4)	174 (9.4)	-3.7	< 0.01*
ISS, Median (IQR)	9 (12)	9 (10)	9 (14)	9 (10)		0.39
ISS, *n* (%)	**5140**	**2591**	**217**	**2332**		
≤ 15	3854 (71.1)	1957 (75.5)	147 (67.7)	1750 (75)	-0.5	0.72
16–25	886 (16.4)	432 (16.7)	43 (19.8)	411 (17.6)	+ 0.9	0.39
26–40	331 (6.4)	160 (6.2)	21 (9.7)	150 (6.4)	+ 0.2	0.75
> 40	69 (1.3)	42 (1.6)	6 (2.8)	21 (0.9)	-0.7	0.03*
Disposition from ED, *n* (%)	**5122**	**2582**	**217**	**2323**		
Ward	3939 (76.9)	1972 (76.4)	155 (71.4)	1812 (78)	+ 1.6	0.22
ICU	965 (18.8)	473 (18.3)	53 (24.4)	439 (18.8)	+ 0.5	0.64
Operating theatre	218 (4.2)	137 (5.3)	9 (4.1)	72 (3.1)	-2.2	< 0.01*
ICU admission, *n* (%)	**5140**	**2591**	**217**	**2332**		0.50
Yes	1213 (23.6)	614 (23.7)	65 (30)	534 (22.9)	-0.8	
No	3927 (76.4)	1977 (76.3)	152 (70)	1798 (77.1)	+ 0.8	
Days in ICU (in days)^**^						
Median (IQR)	9 (14)	9 (14)	6 (14)	9 (14)		0.47
Days in hospital (in days)						
Median (IQR)	8 (12)	8 (13)	8 (12)	9 (12)		0.76
In-hospital mortality, *n* (%)	240 (4.7)	113 (4.3)	13 (6)	114 (4.9)	+ 0.6	0.42

Period 1 = March 25, 2019 – March 24, 2020; Period 2 = March 25 – June 21, 2020; Period 3 = June 22, 2020 – June 21, 2021

*GCS* Glasgow Coma Scale, *ISS* Injury Severity Score, *ED* Emergency Department, *ICU* Intensive Care Unit, *IQR* Interquartile Range

*P*-value: Represent a calculation between period 1 and 3 groups

^*^Significant *P*-value

**Patients who spent 1 day or more in ICU

^$^The lockdown column is just there for completeness

### Risk factors for ICU admission and/ or mortality

The results of unadjusted predictors of in-hospital mortality following injury events are shown in supplementary file, Appendix 1. The results of the multivariable logistic regression models show that age ≥ 60 was associated with an increased risk of mortality for both pre and post easing of restrictions (AOR = 12.85, 95% CIs 3.77–46.74, *p* < 0.01) and (AOR = 10.47, 95% CIs 2.88–43.90, *p* < 0.01), respectively. Patients who required operation on arrival were more likely to die in phase 1 (AOR = 4.65, 95% CIs 2.24–9.65, *p* < 0.01), but not in phase 3. Shorter length of stay in the ICU was significantly associated with mortality in phase 1 (AOR = 0.95, 95% CIs 0.91–0.97, *p* = 0.01) but not in phase 3 (Table 3).

**Table 3:** Multivariable logistic regression model predicting in-hospital mortality

Independent variable	Pre-restrictions Period 1	After easing restrictions Period 3
	Mortality	Mortality
	AOR (95% CI) *P* value	AOR (95% CI) *P* value
Age		
0–14	Ref	Ref
15–29	0.84 (0.31 – 2.43) 0.741	0.93 (0.29 – 3.34) 0.910
30–44	2.43 (0.84 – 7.55) 0.112	1.06 (0.32 – 3.95) 0.927
45–59	1.62 (0.44 – 5.90) 0.461	1.19 (0.28 – 5.12) 0.808
≥ 60	12.85 (3.77 – 46.74) < 0.01	10.47 (2.88 – 43.90) < 0.01
Physiological assessment		
Hospital Pulse/heart rate	1.01 (1.00 – 1.03) 0.021	0.99 (0.98 – 1.01) 0.741
Hospital RR	1.06 (1.00 – 1.11) 0.012	1.02 (0.96 – 1.08) 0.020
Assisted respiration	0.61 (0.25 – 1.44) 0.255	0.25 (0.08 – 0.75) 0.013
Injury type		
Head injury	1.68 (0.77 – 3.72) 0.194	0.89 (0.36 – 2.17) 0.806
Thorax injury	0.87 (0.44 –1.72) 0.701	0.69 (0.33 – 1.43) 0.328
Other trauma	1.00 (0.23 – 3.55) 0.999	0.48 (0.11 – 2.25) 0.358
Mechanism of injury		
Fall	2.28 (1.04 – 4.95) 0.038	1.81 (0.77 – 4.21) 0.168
Burn	9.97 (1.79 – 57.13) 0.01	27.33 (5.12 – 136.68) < 0.01
Mode of arrival		
Government Ambulance	0.85 (0.42 – 1.75) 0.660	0.71 (0.33 – 1.53) 0.391
Private police vehicle	0.32 (0.07 – 1.06) 0.090	0.63 (0.21 – 1.65) 0.371
GCS score	0.74 (0.67 – 0.81) < 0.01	0.815 (0.73 – 0.90) < 0.01
ISS	1.05 (1.02 – 1.08) 0.01	1.06 (1.03 – 1.10) < 0.01
Length of stay in ICU	0.95 (0.91 – 0.97) 0.01	1.01 (0.98 – 1.03) 0.690
Require operation	4.65 (2.24 – 9.65) < 0.01	0.79 (0.24 – 2.21) 0.673

Period 1 = March 25, 2019 – March 24, 2020; Period 2 = March 25 – June 21, 2020; Period 3 = June 22, 2020 – June 21, 2021

*RR* Respiratory Rate, *GCS* Glasgow Coma Scale, *ISS* Injury Severity Score, *ICU* Intensive Care Unit

The mechanisms of injury significantly associated with ICU admission in period 1 were MVCs (AOR = 2.41, 95% CIs 1.72–3.45, *p* < 0.01), motorcycle/cyclists (AOR = 1.68, 95% CIs 1.05–2.68, *p* = 0.03), falls (AOR = 1.93, 95% CIs 1.30–2.92, *p* < 0.01), pedestrians (AOR = 0.70, 95% CIs 0.49–1.02, *p* = 0.05), burns (AOR = 5.76, 3.91–8.66, *p* < 0.01) and penetrating injuries (AOR = 1.69, 1.02–2.78, *p* = 0.04). However, only MVCs (AOR = 2.36, 95% CIs 1.47–3.99, *p* = 0.01) and burns (AOR = 5.56, 95% CIs 3.22–9.97, *p* < 0.01) were significantly associated with ICU admissions in period 3. Furthermore, burns were also significantly associated with in-hospital mortality in both periods, period 1 (AOR = 6.12, 95% CIs 3.18–13.02, *p* < 0.01) and period 3 (AOR = 8.58, 95% CIs 3.28–29.49, *p* < 0.01) (Table 4).

**Table 4:** Adjusted predictors for ICU admission and in-hospital mortality by mechanism of injury

Mechanism of injury, *n* (%)	Pre-restrictions Period 1		After easing restrictions Period 3	
	ICU admission	Mortality	ICU admission	Mortality
	AOR (95% CI) *P* value	AOR (95% CI) *P* value	AOR (95% CI) *P* value	AOR (95% CI) *P* value
Motor Vehicle	2.41 (1.72–3.45) < 0.01	1.31 (0.71–2.71) 0.431	2.36 (1.47–3.99) 0.01	1.54 (0.61–5.19) 0.419
Motorcycle/ cyclist	1.68 (1.05–2.68) 0.030	0.28 (0.04–1.09) 0.107	1.57(0.80–3.09) 0.190	No estimates due to small number
Fall	1.93 (1.30–2.92) < 0.01	1.14 (0.52–2.63) 0.744	1.61(0.87–3.02) 0.133	1.24 (0.35–4.97) 0.742
Pedestrian	0.70 (0.49–1.02) 0.054	0.70 (0.36–1.48) 0.310	0.71 (0.42–1.23) 0.200	0.86 (0.32–2.99) 0.785
Assault	1.60 (0.90–2.79) 0.102	0.49 (0.07–1.90) 0.363	1.39 (0.61–3.03) 0.419	0.54 (0.03–3.76) 0.586
Burn	5.76 (3.91–8.66) < 0.01	6.12 (3.18–13.02) < 0.01	5.56 (3.22–9.97) < 0.01	8.58 (3.28–29.49) < 0.01
Penetrating	1.69 (1.02–2.78) 0.040	0.53 (0.12–1.78) 0.347	1.18 (0.53–2.56) 0.675	1.54 (0.30–7.19) 0.582

## Discussion

To our knowledge, this is the first study that seeks to understand the impact of easing of COVID-19 lockdown restrictions on injuries in Saudi Arabia using clinical data and one of the first such studies internationally. Accessing data from a major trauma centre, this study explored a large number of injury variables such as demographics, mechanisms of injury, severity of injury and patient outcomes before and after easing of COVID-19 lockdown restrictions. Our study has observed a change in the demographics, the mechanisms of injury and the outcomes of the trauma after easing of restrictions. A reduction of 5.2% was observed in the overall volume of all injury admissions during the first 12 months post lifting of lockdown restrictions. A reduction in the proportion of cases resulting in an ICU admission and patients requiring surgery on arrival was seen in the post easing of restrictions period. This decrease might be attributed to a lower number of patients in the post restrictions period presenting with severe trauma and/or decreased level of consciousness.

Despite this, with respect to the risk of in-hospital mortality according to the mechanism of injury, there was no difference between the pre and post lockdown periods; however, we identified that motorcycle/pedal cycle, pedestrian, fall, and penetrating injuries were associated significantly with ICU admission in period 1 but not in period 3. Furthermore, we found that there was a 5% decrease in the odds of mortality for each additional day in ICU in period 1. We also witnessed a general decrease in the LOHS post easing of COVID-19 restrictions, but there was no change in the length of ICU stay in the two periods. It should be noted that the change in LOHS could be the result of COVID-19 infections, as hospitals possibly limited admissions during the COVID-19 outbreak and/or altered patient preferences, in addition to hastening patient discharge in order to limit potential exposure to the virus [19, 20].

Not surprisingly, the rates of injury at home and incidences of assault/violence increased significantly during the post lockdown period. Our findings are consistent with other studies that demonstrate the adverse psychological impact of COVID-19 on the global population [21, 22] which may have led to an increase in violence-related injuries [23, 24]. This psychological impact is expected to be long-lasting, persisting beyond the lockdown phase, because people may have lost family members, jobs or businesses during the virus outbreak [25]. These findings suggest that the causes of increased violence-related injuries should be investigated in future studies.

In this study, 45.6% of the patients arrived at the hospital by government ambulance; such patients are more likely to be referral patients because Saudi Red Crescent is the primary prehospital care provider in the country. However, transferring of patients between hospitals is usually undertaken by the Ministry of Health (MOH) ambulances (government ambulances). As a mode of arrival, there was no difference between Red Crescent ambulances and MOH ambulances between periods 1 and 3; however, a reduction in private vehicle/ police vehicle arrival was noted in period 3. One potential explanation for this decrease is the widespread COVID-19 infections, as the general public as well as the policymakers were cautious about transporting patients using their vehicles. There has also been a notable decrease in the ISS, suggesting that the overall injury burden was slightly lower in the pre as well as post lockdown groups.

In Saudi Arabia, road injuries are a major public health problem [9, 11]. With regard to the different etiologies of road trauma, although road trauma decreased post easing of restrictions, it is still the leading cause of injury across the three periods, causing around 50% of total trauma. We also reviewed the MOH statistics and indicators to identify the rate of road traffic mortality. The mortality was 5,754 cases in 2019 and 4,618 cases in 2020, which further shows that the burden of road traffic injury was significant even during the country-wide lockdown [26]. Given that an all-inclusive trauma system plays an essential part in decreasing mortality arising from road injuries [27, 28], there are opportunities to extend and strengthen the trauma care system in Saudi Arabia [9]. Moreover, injury prevention needs a robust community programme and legislation around road safety measures, including strict enforcement that can decrease the burden of traumatic injuries in the country.

Interestingly, there has been a significant increase in burn-related injuries both during and post lockdown periods. This could be the result of extended home schooling for several months after easing of lockdown restrictions as children spent more time at home [3]. We also found that the incidence of injury at home increased significantly in the post lockdown group. Hence, we are led to believe that lockdown prompted more cooking and dining activities at home, which is another possible contributor to the increased burn-related injuries.

With respect to the mechanism of injury, our study found that the most significant reduction was identified in the pedestrian population. Primary school students (years 1–6) are yet to return to on-site learning as of October 2021, which is a possible explanation for this significant drop, particularly when pedestrian injuries represent 71% of the total road injuries among children in Saudi Arabia [29]. Therefore, promotion of road safety is recommended around school zones together with traffic law enforcement.

This study has several limitations. First, we compared a period of 12 months before and a period of 12 months after lifting of COVID-19 restrictions; the duration of these two periods were marginally different (by three months) because we compared April 2019–March 2020 (phase 1) to July 2020–June 2021 (phase 2). We were interested in revealing the early impact of lifting policy restrictions. Nevertheless, our study also observes a one-year period of injury data covering all seasons. Second, although most of the restrictions were lifted by the end of June 2020, some protocols were in place for a longer time, such as limits on participants for social and religious gatherings. However, no lockdown policies or restrictions on travelling between regions were in effect beyond July 2020. Third, the population included in this study represents a single-centre experience. Thus, this limits the generalisability of our findings to different cities and regions in Saudi Arabia.

## Conclusion

This retrospective study of a major trauma centre demonstrated a significant change in the mechanisms of injury and outcomes of the trauma population. MVC continues to be the leading cause of injury in Saudi Arabia. These findings suggest that the causes of increased violence-related injuries should be investigated. Although the overall injury cases decreased in the first 12 months after easing of COVID-19 restrictions, injury cases rebounded towards pre-lockdown levels with increased in-hospital mortality. Injury prevention needs a robust community programme and legislation with respect to road safety measures together with strict enforcement to decrease the burden of traumatic injuries in Saudi Arabia.

## Supplementary Information

**Additional file 1: Appendix 1.** Univariatepredictors of in-hospital mortality following injury by different variables. 

## Data Availability

The datasets used during the current study are available from the corresponding author on reasonable request.

## Abbreviations

COVID-19: Coronavirus Disease 2019
WHO: World Health Organization
MVC: Motor Vehicle Crashes
KSMC: King Saud Medical City
STAR: Saudi TraumA Registry
ED: Emergency Department
ICU: Intensive Care Unit
SBP: Systolic Blood Pressure
HR: Heart Rate
RR: Respiratory Rate
SD: Standard Deviation
DV: Dependent Variable
IV: Independent Variables
AOR: Adjusted Odds Ratios
CIs: Confidence Intervals
ISS: Injury Severity Score
GCS: Glasgow Coma Scale
LOHS: Length Of Hospital Stay
