# Supplementary material for: Impact of easing COVID-19 lockdown restrictions on traumatic injuries in Riyadh, Saudi Arabia: one-year experience at a major trauma centre
Source: BMC Public Health. 2023 Jan 4;23:22. doi: 10.1186/s12889-023-14981-9 (PMC9812537; doi:10.1186/s12889-023-14981-9)
Supplement: Supplementary file 1 — Additional file 1: Appendix 1. Univariatepredictors of in-hospital mortality following injury by different variables. [file 12889_2023_14981_MOESM1_ESM.docx]

**Supplementary file, Appendix 1.**

Univariate predictors of in-hospital mortality following injury by different variables

| **Independent variable** | **Pre-restrictions**  **Period 1** | **After easing restrictions**  **Period 3** |
| --- | --- | --- |
|  | Mortality | Mortality |
|  | OR (95% CI) *p* value | OR (95% CI) *p* value |
| Age  0–14  15–29  30–44  45–59  ≥60 | 1.57 (0.88 – 2.63) 0.11  0.61 (0.40 – 0.91) 0.02  0.85 (0.53 – 1.31) 0.47  1.36 (0.77 – 2.26) 0.25  1.88 (1.06 – 3.12) 0.02 | 1.23 (0.61 – 2.24) 0.52  0.89 (0.59 – 1.31) 0.55  0.87 (0.57 – 1.30) 0.51  0.63 (0.30 – 1.16) 0.17  2.14 (1.26 – 3.47) <0.01 |
| Gender  Male  Female | 1.03 (0.63 – 1.77) 0.91  0.97 (0.56 – 1.58) 0.91 | 0.90 (0.56 – 1.54) 0.69  1.10 (0.65 – 1.79) 0.69 |
| Physiological assessment |  |  |
| Pulse/heart rate at scene | 1.02 (0.99–1.03) 0.03 | 1.02 (0.99–1.04) 0.10 |
| ED systolic BP | 0.98 (0.96–0.98) <0.01 | 0.99 (0.98–1.00) 0.07 |
| ED pulse/heart rate | 1.02 (1.01–1.03) <0.01 | 1.02 (1.01–1.02) <0.01 |
| ED respiration rate | 1.09 (1.04–1.14) <0.01 | 1.12 (1.06–1.17) <0.01 |
| Respiratory Assistance requirement | 0.06 (0.03–0.09) <0.01 | 0.05 (0.03–0.07) <0.01 |
| Type of injury |  |  |
| Head | 4.53 (3.08 6.71) <0.01 | 2.92 (1.99–4.27) <0.01 |
| Neck | 6.03 (1.69–16.93) 0.01 | 1.63 (0.08–8.37) 0.64 |
| Thorax | 3.93 (2.68–5.77) <0.01 | 2.65 (1.79–3.88) <0.01 |
| Abdomen and pelvic | 3.85 (2.44–5.91) <0.01 | 1.02 (0.49–1.89) 0.94 |
| Upper extremities | 0.85 (0.53–1.30) 0.48 | 0.58 (0.33–0.94) 0.03 |
| Lower extremities | 0.88 (0.59–1.28) 0.50 | 0.44 (0.28–0.66) <0.01 |
| Other trauma | 3.39 (2.04–5.40) <0.01 | 5.00 (3.18–7.68) <0.01 |
| Injury time | 1.51 (0.85–2.75) 0.16 | 1.79 (1.01–3.25) 0.04 |
| Mechanism of injury |  |  |
| Road trauma | 1.09 (0.75 – 1.60) 0.66 | 0.73 (0.49 – 1.07) 0.11 |
| Fall | 0.51 (0.31–0.80) <0.01 | 0.56 (0.34–0.86) 0.011 |
| Burn | 4.78 (2.80–7.82) <0.001 | 6.32 (4.04–9.70) <0.01 |
| Mode of arrival |  |  |
| By Government Ambulance | 3.4 (2.29–5.09) <0.01 | 2.77 (1.87–4.13) <0.01 |
| By Private ambulance | 2.62 (0.98–5.77) 0.02 | 2.7 (0.63–7.95) 0.11 |
| By private or police vehicle | 0.15 (0.05–0.34) <0.01 | 0.30 (0.12–0.60) <0.01 |
| By Red Crescent ambulance | 0.70 (0.42–1.10) 0.14 | 0.57 (0.33–0.92) 0.02 |
| ICU admission | 8.24 (5.50–12.47) <0.01 | 22.13 (13.96–36.59) <0.01 |
| Operation | 8.37 (5.17–13.26) <0.01 | 2.18 (0.89–4.55) 0.05 |
| First GCS Total | 0.73 (0.69–0.76) <0.01 | 0.75 (0.71–0.78) <0.01 |
| Injury Severity Score ISS | 1.10 (1.08–1.11) <0.01 | 1.09 (1.07–1.11) <0.01 |
| Length of stay in ICU | 1.03 (1.01–1.04) <0.01 | 1.07 (1.05–1.08) <0.01 |
| Length of hospital stay | 1.00 (0.98–1.00) 0.53 | 1.01 (1.00–1.02) <0.01 |
